# COVID-19 Diagnosis: A Review of Rapid Antigen, RT-PCR and Artificial Intelligence Methods

**DOI:** 10.3390/bioengineering9040153

**Published:** 2022-04-03

**Authors:** Raphael Taiwo Aruleba, Tayo Alex Adekiya, Nimibofa Ayawei, George Obaido, Kehinde Aruleba, Ibomoiye Domor Mienye, Idowu Aruleba, Blessing Ogbuokiri

**Affiliations:** 1 Department of Molecular and Cell Biology, Faculty of Science, University of Cape Town, Cape Town 7701, South Africa; arlrap001@myuct.ac.za; 2 Department of Pharmacy and Pharmacology, School of Therapeutic Science, Faculty of Health Sciences, University of the Witwatersrand, Johannesburg, 7 York Road, Parktown 2193, South Africa; 2272680@students.wits.ac.za; 3 Department of Chemistry, Bayelsa Medical University, Yenagoa PMB 178, Bayelsa State, Nigeria; ayawei4acad@gmail.com; 4 Department of Computer Science and Engineering, University of California, San Diego, La Jolla, CA 92093-0404, USA; 5 School of Computing and Mathematical Sciences, University of Leicester, Leicester LE1 7RH, UK; 6 Department of Electrical and Electronic Engineering Science, University of Johannesburg, Johannesburg 2006, South Africa; ibomoiyem@uj.ac.za (I.D.M.); 219098852@stu.ukzn.ac.za (I.A.); 7 Department of Mathematics and Statistics, York University, Toronto, ON M3J 1P3, Canada; blessogb@yorku.ca

**Keywords:** COVID-19, SARS-CoV-2, artificial intelligence, machine learning, deep learning, molecular diagnosis

## Abstract

As of 27 December 2021, SARS-CoV-2 has infected over 278 million persons and caused 5.3 million deaths. Since the outbreak of COVID-19, different methods, from medical to artificial intelligence, have been used for its detection, diagnosis, and surveillance. Meanwhile, fast and efficient point-of-care (POC) testing and self-testing kits have become necessary in the fight against COVID-19 and to assist healthcare personnel and governments curb the spread of the virus. This paper presents a review of the various types of COVID-19 detection methods, diagnostic technologies, and surveillance approaches that have been used or proposed. The review provided in this article should be beneficial to researchers in this field and health policymakers at large.

## 1. Introduction

The recent incident of the novel coronavirus (SARS-CoV-2) in Wuhan, China, and its spread globally has impacted the world’s economy. So far, the virus has claimed more than 5 million lives and infected over 278 million people worldwide as of 27 December 2021 [1]. The emergence of different variants shows that the fight against the deadly and infectious viruses is far from over [1]. It also shows how swiftly new infectious diseases might emerge and spread while wrecking global economic havoc. The viral aetiology of coronavirus disease 2019 (COVID-19) is SARS-CoV-2, which has unexpectedly increased the need for clinical knowledge and information, epidemiological investigations, and quick diagnostic technology.

It is well known that quick, efficient, and ultrasensitive detection of SARS-CoV-2 is crucial for epidemic prevention and containment [2,3]. As a result, there has been worldwide demand for knowledge on SARS-CoV-2 diagnostic and surveillance technologies. With accurate diagnostics in place, health workers may decide where and how to allocate resources and efforts to effectively isolate and treat patients. This mechanism can slow the spread of infectious diseases and minimise mortality. Thus, all of the tools required for the rapid detection of SARS-CoV-2 are extremely useful to frontline healthcare workers and policymakers working together to alleviate the disease’s devastation and limit its spread. Since the emergence of COVID-19, many methodologies have been used worldwide to identify and diagnose the virus. The approaches reported include whole-genome sequencing, electron microscopy, and different computed tomography (CT) imaging based methods, which were initially employed to screen for and detect SARS-CoV-2 [4,5].

Meanwhile, the availability of known diagnostic tools for SARS-CoV-1 (from the 2002 SARS outbreak) was helpful in the diagnosis of COVID-19. These tools are currently performing critical roles in detecting and controlling the spread of COVID-19. For example, the transmission electron microscopy (TEM) was employed to determine the morphology of the SARS-CoV-2 virus [6]. The virus’s identification was confirmed via genome sequencing [7,8], and the sequencing data was used in the development of primers and probes for polymerase chain reaction (PCR) [9]. It is worth noting that whereas the SARS-CoV-1 identification took about five months in 2002, the same procedures were employed to identify SARS-CoV-2 within a few days [10]. Recently, artificial intelligence (AI) has shown great potential in detecting diverse diseases [11,12,13]. Some AI-based methods have been proposed for COVID-19 detection, tracking, and treatment [14,15,16,17]. Deep learning, a subfield of AI that is based on artificial neural networks [18], has been applied to learn and analyze the lung regions using CT images and chest radiographs (X-ray) in order to detect COVID-19 [19,20].

This study presents a detailed review of the various types of detection methods, diagnostic technologies, and surveillance approaches that have been used or recently proposed in the fight against COVID-19. Holistically, this will aid decision making by researchers and policymakers. The rest of this paper is structured as follows: The current and emerging COVID-19 diagnostic tests are presented in Section 2. Section 3 discusses real-time reverse transcriptase-polymerase chain reaction, and rapid antigen detection test is presented in Section 4. Section 5 presented a detailed application of AI techniques for COVID-19. Section 6 highlights some contributions of AI in the fight against COVID-19. Finally, the paper is concluded in Section 7.

## 2. Current and Emerging COVID-19 Diagnostic Tests

Nucleic acid tests and computer tomography (CT) scans were earlier used to diagnose COVID-19. At the start of the COVID-19 epidemic in China, syndromic testing using CT scans was predominantly employed to diagnose and examine the virus [21]. However, molecular technology is more suitable for detecting the virus than syndromic examination and CT scans because it can target and detect specific infections. However, due to the need for a more effective and real-time diagnosis of COVID-19, researchers and scientists have created several tools summarised in Table 1. These technologies are divided into three categories: nucleic acid testing, protein testing, and point-of-care (POC) testing.

**Figure 1:**
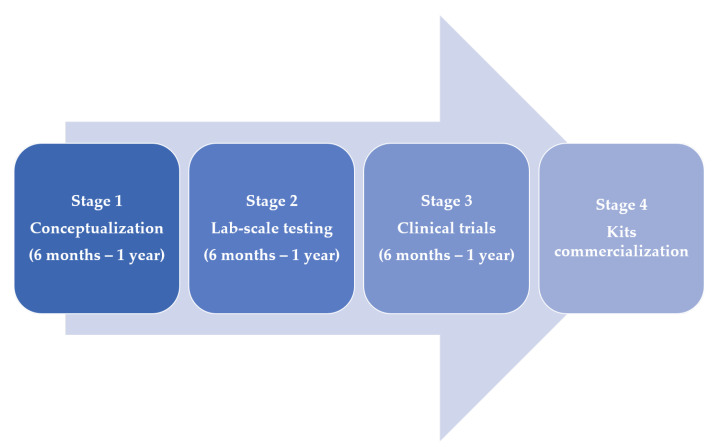
The diagram depicts four pipeline for diagnostics technologies development. In most cases, stages 1 and 2 are designed and achieved by researchers while stages 3 and 4 typically involve commercial transfer to companies.

Figure 1 also depicts the stages of development of diagnostic tests used thus far. As can be observed, most of the approaches are still in the proof-of-concept stage. The majority of the proposed diagnostics technologies are likely to enter the commercial phase and be applied for disease detection in the future. Notably, it remains unpredictable if the vast knowledge gathered from the various variants seen so far can be generalised for investigating future variants of the COVID-19 virus. For example, to mitigate the widespread of the new variant (Omicron) that was first confirmed on 9 November 2021 [22], testing will be paramount. And to achieve this, less expensive, sensitive, user-friendly, and point-of-care kits will be required. Such kits will ultimately reduce a surge in cases as people can self-test and isolate themselves accordingly.

## 3. Real-Time Reverse Transcriptase-Polymerase Chain Reaction

In recent years, nucleic acid detection-based techniques have become a reliable and rapid approach for detecting viruses. Precisely, the polymerase chain reaction (PCR) has gained popularity and is considered the gold standard for diagnosing some variants of viruses and is characterised by rapid detection, high sensitivity and specificity [33]. Based on those above, real-time reverse transcriptase-PCR (RT-PCR) has gained much interest in detecting SARS-CoV-2 due to its specific and easy qualitative assay. To achieve its aim, the RT-PCR involves the reverse transcription of the virus RNA into complementary DNA (cDNA) strands, followed by the amplification of certain regions of the cDNA. The amplified cDNA of the virus is targeted, and a section of the SARS-CoV-2 genome is amplified via PCR [34].

The Center for Disease Control and Prevention (CDC) presented a recent study using the Fulgent COVID-19 RT-PCR test to detect COVID-19 [35]. A total of 2039 patients admitted to the emergency department in a California hospital between June and August of 2020 participated in the study, and the RT-PCR test obtained a specificity above 99%. Another study used the RT-PCR test to detect COVID-19 in samples from 323 patients, and metrics such as sensitivity and specificity, with confidence intervals (CI) that indicate the test results’ statistical significance, were used in the study [36]. The RT-PCR obtained a sensitivity of 94.7% (95% CI 74.0 to 99.9%) and a specificity of 100% (95% CI 94.9 to 100%). To date, the RT-PCR test is the most commonly used test for detecting COVID-19, and several studies have stated its robustness and reliability compared to other testing methods [37,38,39,40].

The SARS-CoV-2 RNA is extracted and diluted with a master mix containing both forward and reverse primers, nuclease-free water, a fluorophore-quencher probe and a reaction mix (magnesium, transcriptase, nucleotides, polymerase, and additives). The extracted viral RNA and master mix are amplified in a PCR thermocycler, and the respective incubation parameters are set to run the assay. During the assay, the cleavage of the fluorophore-quencher probe results in a fluorescent signal detected by the thermocycler and amplification is recorded in real-time. The primary method of this diagnostic test involves the collection of samples from the upper respiratory of a person via nasal and oropharyngeal swabs. Notably, swabs from the nose and throat can produce false-negative results if not done correctly. Hence, the person performing the test must be familiar with the upper respiratory anatomy [41]. False-negative tests may also occur due to mutation in the real-time RT-PCR primer and probe-target segments of the virus genome [33]. Others may include but are not limited to sample cross-talk, and hitches with software may reduce the sensitivity of PCR reaction. More so, the commercially available RT-PCR kits are only operated in well-equipped laboratories due to the specialised tools and instruments used for the reactions coupled with safety reasons, thereby limiting the number of tests that can be performed daily [42]. Several nucleic acid amplification tests have been authorized by the U.S. Food and Drug Administration (FDA) [43]. These kits have been used to analyze samples from the nose, throat, bronchoalveolar lavage and sputum, and the sensitivity of some are higher than RT-PCR. For example, loop-mediated isothermal amplification (LAMP) can rapidly amplify the viral RNA at a single temperature whilst providing a reliable diagnosis [44]. Some of these tests can be self-administered at home or in laboratories. Indeed, antigen tests targeting various virus proteins could be vital to support the currently available RT-PCR kits and speed up detection.

## 4. Rapid Antigen Detection Test

A rapid antigen test (RAT), also known as an antigen rapid test (ART), or a rapid antigen detection test (RADT), or simply a rapid test, is a type of rapid diagnostic test that immediately identifies the absence or presence of an antigen for point-of-care testing. Rapid tests are a form of lateral flow device that detects antigens, as opposed to other medical tests that detect nucleic acid (nucleic acid tests) or antibodies (antibody tests), of either point-of-care types or laboratory. Usually, it is widely used to detect SARS-CoV-2, the virus that causes COVID-19. Rapid tests often have fast turnaround times of less than 5 to 30 min, involve minimal training or equipment, and offer considerable cost benefits, and may be employed in decentralised testing; and they have the potential to boost testing procedures [45].

Meanwhile, Gremmels et al. [45] studied the potential of PanbioTM COVID-19 antigen rapid test and its diagnostic performance, which is yet to be fully confirmed. According to their results, the PanbioTM COVID-19 Ag rapid test successfully detects SARS-CoV-2 infected people with a significant viral load in nasopharyngeal samples in a population of participants dwelling in a community with mild respiratory tract infection, and this test has a 100 percent specificity. Though the sensitivity is lesser than that of RT-qPCR, all false-negative rapid test findings were attributed to low viral loads in nasopharyngeal samples. The study concluded that because the PanbioTM COVID-19 Ag rapid test has a lower sensitivity, RT-qPCR would be the appropriate diagnostic test for clinical applications in a hospital environment.

However, for community monitoring of SARS-CoV-2, this rapid antigen test accurately and quickly identifies persons with a high risk of continued transmission. In the future, it might be an essential tool in our testing strategy to prevent SARS-CoV-2 transmission. In another research, Torres et al. [46] studied the performance of the PanbioTM COVID-19 Ag rapid test device to identify and detect SARS-CoV-2-infected asymptomatic peoples. The study discovered that the Panbio test has limited sensitivity in asymptomatic COVID-19 patients close contacts, especially non-household contacts. Notably, the authors believe that determining the best time to collect upper respiratory tract samples in this group is critical for pinpointing test sensitivity [46]. However, a low sensitivity test such as this is not suitable for detecting COVID-19, considering the impact a false-negative test result can cause.

Furthermore, Mak et al. [47] studied the specificity and sensitivity of COVID-19 Ag rapid tests such as SARS-CoV-2 detection using the rapid antigen detection kit from the WHO Emergency Use List. The findings from the study revealed that the rapid antigen detection kit was 100 times less sensitive than RT-PCR. The clinical sensitivity of the rapid antigen detection test for identifying specimens from COVID-19 patients was 68.6%. A multicenter study was also conducted by Merino et al. [48] also conducted a multicenter study to evaluate the PanbioTM COVID-19 rapid antigen-detection test for SARS-CoV-2 infection diagnosis.

## 5. Artificial Intelligence and COVID-19

### 5.1. Machine Learning

Machine learning (ML) has shown high performance in several image processing applications such as image analysis [49], image segmentation and classification [50], and medical imaging [11,51]. With the recent outbreak of the COVID-19 pandemic, ML offers a great potential for accurate and fast detection of COVID-19 from computed tomography (CT) and chest radiographs (CXR) images.

As we learn more about the natural history of COVID-19, it has become apparent that the disease progresses in stages. The need to pre-empt deterioration and personalise preventative interventions has emerged as a priority [52]. Currently, imaging research has focused on diagnosis based on appearances once the disease has progressed. Early detection of the infection, when the initiation of appropriate therapy is likely to be most effective, would be more helpful. CT also has a well-established role as a tool to detect several diseases, particularly when combined with clinical data. This finding is essential in COVID-19 detection; because the primary concern for healthcare providers is becoming overwhelmed by patients requiring intensive care and ventilatory support, accurate prognostication is a more pressing clinical problem than diagnosis [53]. For COVID-19, training a model to predict outcomes such as the need for mechanical ventilation, intensive care unit admission, and mortality could have a considerable clinical effect [54,55,56].

Since the pandemic, several studies have been conducted on how ML can be used to detect and diagnose COVID-19. The systematic review in [57] critically examined the methodologies used in 29 studies that focused on ML and COVID-19. Of these 29 studies, seven were based on conventional ML algorithms, 20 on deep learning techniques, and two used deep learning and traditional ML techniques together. Most of the studies, i.e., 23, address the detection of COVID-19, while six focused on building systems for prognostication. The findings in Roberts et al. [57] showed that none of the developed models in the 29 papers has any potential for clinical use because of their methodology’s underlying biases and flaws. An ML model was developed in [58] for screening potential neutralising antibodies discovered for the COVID-19 virus. The model was developed to check synthetic antibodies to detect antibodies that potentially inhibit SARS-CoV-2. The study used 14 different types of viruses and developed models using graph featurisation with different ML algorithms such as support vector machine, random forest, and logistic regression. The model’s out-of-class predictions for covid and influenza were 100% and 84.61%, respectively; this shows the model’s robustness in neutralising predicted antibodies for the SARS-CoV-2. To understand the spread of the virus in the top five affected countries (USA, India, Brazil, Peru, and Russia) as of 10 July 2020, Hazarika and Gupta [59] proposed a new approach based on a random vector functional link (RVFL) network. RVFL was hybridised with a wavelet-coupled RVFL network and 1-D discrete wavelet transform. Hazarika and Gupta [59] concluded that the wavelet-based hybrid models can be useful in the fight against COVID-19.

Elaziz et al. [60] introduced an ML technique that uses fractional multichannel exponent moments (FrMEMs) to extract features from X-ray images. The technique classified chest X-ray images into two classes, i.e., COVID 19 patients and non-COVID-19 patients. The proposed method achieved an accuracy of 96.09% in predicting the COVID 19 class and 98.09% in the non-COVID-19 class. Meanwhile, India is among the countries worse hit by the COVID-19 pandemic [61]. During the pandemic surge in that country, an ML forecasting model was proposed in [62] to assist in predicting the spread of the virus. The authors in Sujath et al. [62] used multilayer perceptron, linear regression, and vector autoregression on datasets obtained from Kaggle to understand the fast-spreading virus across the country, i.e., confirmed, death, and recovered cases across India. The review in Kushwaha et al. [63] discussed the importance of ML in the fight against the COVID-19 pandemic. The research examined several papers that addressed how different ML models could have assisted in detecting the virus. The review concluded that ML could be used for COVID-19 diagnosis, precise and personalised patient treatment, patient behaviour analysis, and future symptoms prediction. A hybrid ML model based on a multilayer perceptron algorithm and adaptive network-based fuzzy inference system was used to predict a patient mortality rate [64]. The dataset used was collected in Hungary, and the model validation was performed for nine days with good results. ML-based CT analysis has shown to be a promising screening medium for COVID-19 and has outperformed viral real-time PCR testing [65,66].

### 5.2. Deep Learning

Although machine learning powers many aspects of societal applications from social media networks to consumer products and devices, supported by cameras and smartphones [67]. However, conventional machine learning techniques are not well-suited for some modern societal applications [68]. The advent of deep learning, a subset of machine learning, has found applications in many areas, such as object and speech detection, drug and genomics, and natural language processing systems [67,69].

**Figure 2:**
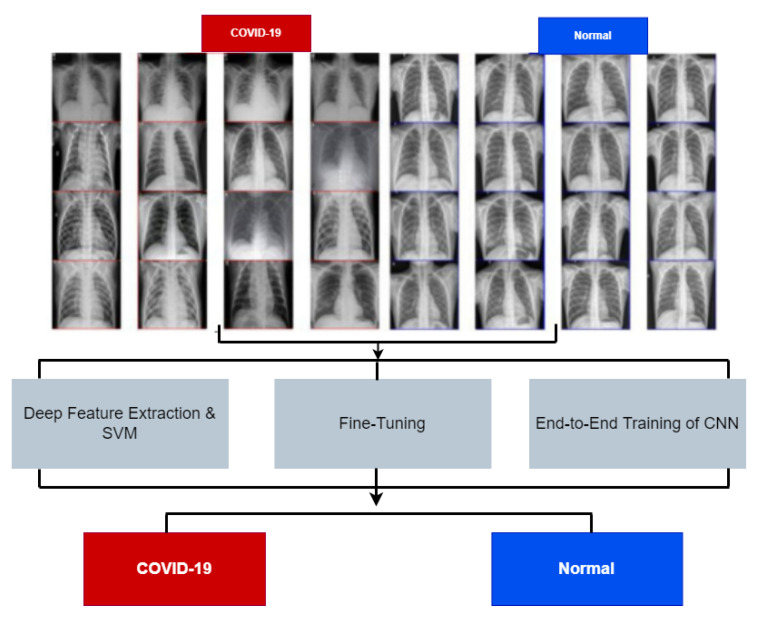
Deep learning model for COVID-19 detection [78].

Since the outbreak of COVID-19, deep learning algorithms have been widely used to understand and forecast the disease pattern [70,71]. Several attempts have been made to utilise these algorithms to estimate and forecast the future spread of the disease [72]. A review by [70] on six deep learning algorithms used for disease forecasting shows popular algorithms such as Recurrent Neural Network (RNN), Long Short-Term Memory (LSTM), Bi-directional (Bi-LSTM), Variational Auto Encoder (VAE), and Gated recurrent units (GRUs) have been used on time-series data for predicting newly affected and recovered cases of COVID (See Figure 2).

These models are based on time-sensitive analysis (temporal) and possess attractive modelling features. All these algorithms are based on RNN [73,74]. Meanwhile, RNNs possess less memory and are not suited for time dependencies in historical data. LSTMs were designed to mitigate many of the dependency issues of RNN, and they contain three gates for controlling information flow—input, forget, and output gates. Bi-LSTM enhances the capabilities of the LSTM and retains the option of being reconstructed to a backward context [73]. The GRU models are an alternative to LSTM. GRUs was created to optimise the performance of LSTM models, and VAE models are based on generative modelling and extend the capabilities of RNNs.

Other works in literature have explored the convolutional neural networks (CNN) [75,76,77]. CNN is a type of artificial neural network that has been widely used for medical imaging analysis. It has multiple layers to process data at less computational power with higher accuracy. A pre-trained CNN was used in classifying X-ray images to uncover healthy and non-healthy chest scans [78]. These pre-trained models include ResNet18, ResNet50, ResNet101, VGG16, and VGG19. The ResNet50 model was trained alongside the support vector machine, which was found to have achieved higher accuracy. Other CNN variants have been created to join the fight against COVID, which include CoVNet-19 [79], Res-CovNet [80], and MTU-COVNet [81].

### 5.3. COVID-19 Datasets

Data is essential in all machine learning models and applications [82]. If data is made publicly available, ML algorithms can help in reducing the spread of COVID-19. Therefore, the first step in designing a COVID-19 detection model is data collection. Having ML-based COVID-19 diagnosis from X-rays and CT scans will lower the challenge on the short supplies of reverse transcriptase-polymerase chain reaction (RT-PCR) test kits. Epidemiological and statistical analysis of reported covid cases can also be useful in understanding the relationship between virus transmission and human mobility. Social media data can also provide socio-economic and sentiment analysis for governments and policymakers in the current pandemic. Therefore, data is essential for the effective implementation of models to fight and reduce the spread of the virus.

Most available datasets were private at the beginning of the pandemic due to privacy issues. However, several of these datasets were recently made open to researchers [83]. Meanwhile, most researchers have focused on using CT image datasets for their diagnosis due to the time consumed and low sensitivity associated with the standard COVID-19 diagnosis methods, such as the RT-PCR and CXR [84,85,86,87,88]. This has made both CT and chest X-ray diagnostic imaging modalities quickly produce large volumes of data on COVID-19, which has enabled the development of machine learning models for detecting and diagnosing the virus.

At the start of the COVID-19 pandemic, radiologists were extremely busy with little bandwidth to read many CT scans timely. Also, radiologists in developing countries may not be well equipped to recognise COVID-19 from CT scans since the disease was relatively new at the time. Therefore, in order to accurately detect the virus, several deep learning methods were developed to screen COVID-19 from CTs [65,89,90,91]. COVID-CT, an open-sourced dataset, was introduced in [92]. The dataset comprises 349 positive COVID-19 CT images and 463 negative COVID-19 CTs. The dataset was extracted from reported CT images in 760 bioRxiv and medRxiv preprints about COVID-19. Experiments on the dataset showed that COVID-CT is efficient in developing an AI-enabled model to diagnose COVID-19. The authors [92] further used the dataset to build self-supervised and multi-task learning, which achieved an area under the receiver operating curve (AUC) of 0.98, F1-score of 0.90, and accuracy of 0.89. Table 2 summarises some notable COVID-19 datasets in the literature, the techniques used in building the ML model, and the obtained results. The metrics used in evaluating the performance includes accuracy (ACC), sensitivity (Sen), and specificity (Spe).

Afshar et al. [93] proposed COVID-CT-MD, a new COVID-19 dataset. The dataset also consisted of healthy and participants infected by Community-Acquired Pneumonia (CAP). The COVID-CT-MD consists of 169 chest CT scans of positive patients, 60 patients with CAP, and 76 patients that do not have either covid or CAP. The results obtained from COVID-CT-MD showed that the dataset could advance the application of ML and DL in diagnosing COVID. Ref. [85] developed an open-source SARS-CoV-2 CT scan dataset to encourage the development of AI techniques for detecting COVID-19 through the analysis of their CT scan. SARS-CoV-2 CT contains 2482 CT scans, with 1252 positive and 1230 non-infected cases. The dataset is made up of actual patient CT scans collected in Sao Paulo, Brazil. Using the eXplainable Deep Learning technique (xDNN), Angelo and Soares [85] achieved an F1-score of 97.31%, which is promising. Furthermore, in light of understanding how chest X-ray images can assist in diagnosing COVID-19, Hall et al. [94] obtained 320 chest X-rays of bacterial and viral pneumonia and 135 chest X-rays of positive COVID-19 patients. These datasets were used with a pre-trained deep convolutional neural network (DCNN). The model achieved an accuracy of 91.24% and an AUC of 0.94. Another notable application of ML in the COVID-19 era is contact tracing, which has yielded excellent results [95].

## 6. Notable Contributions of AI in the Fight against COVID-19

### 6.1. AI for COVID-19 Tracking and Dashboarding

Since the outbreak of COVID-19, there have been concerted efforts towards tracking and predicting its debilitating effect across many nations [96,97,98]. These unified efforts have led AI researchers to utilise predictive modelling for forecasting actual and expected spread and reporting on open data dashboards, thereby supporting the efforts of healthcare professionals.

One of the foremost real-time dashboards used for COVID-19 tracking was developed by the Center for Systems Science and Engineering (CSSE) at the John Hopkins University [99]. The CSSE platform has effectively tracked recoveries, death, and new cases worldwide. This platform aimed to provide stakeholders, such as public authorities, researchers, and the broader public, with an interactive interface to track the virus in real-time [100]. Similar platforms have emerged to unify monitoring and prediction efforts. These dashboards include the Center for Disease Control and Prevention (CDC), COVID-19 Data Tracker, Microsoft Bing’s COVID-19 tracker dashboard, the BBC, New York Times, HealthMap, and Upcode. Other notable platforms include nCoV2019, 1point3arces, China’s Baidu, South Korea’s KSIC, Time’s Coronavirus Map, NPR, and Worldmapper [101].

Countries within the Global South have intensified efforts to create dashboards for monitoring and predicting COVID-19 [102,103,104]. The African Union Centre for Disease Control, a public health agency for member states, created the African CDC Dashboard to provide updates on the COVID-19 crisis within the region [105]. Another popular example is the COVID-19 ZA South Africa Dashboard, developed at the University of Pretoria by the Data Science for Social Impact Research Group (DSFSI). In South America, especially in Panama, a dashboard termed the COVID-19 Open data was developed to track and predict cases [106]. Saudi Arabia launched its own dashboard in the Middle East to enable public authorities to monitor and combat COVID-19 cases. Table 3 shows the summary of COVID-19 dashboards discussed in this research, and they are classified according to the source, name of dashboard, country, its purpose, coverage, and accessible medium. However, this list is not exhaustive.

### 6.2. AI for COVID-19 Diagnosis and Forecasting

Recently, researchers have intensified efforts to combat the threats posed by COVID-19 using different techniques. Several AI initiatives are in continuous development to detect COVID-19 infections, assisting healthcare professionals. A study by [113] used the Random Forest (RF) algorithm to extract eleven key blood indices to accurately identify traces of the COVID-19 virus on patients’ blood test data. The study showed that the RF algorithm extracted the features, enabling the predictive model to achieve an accuracy of 96.7%. Similarly, Tang et al. [114] applied the RF algorithm on chest CT images and identified features of COVID-19. The study reported an accuracy of 87.5% and an AUC of 91%.

Yan et al. [115] proposed a method to detect COVID-19 using the extreme gradient boosting (XGBoost) classifier. The XGBoost classifier was trained with samples from 485 infected patients, and it achieved excellent performance. In another research, Bertsimas et al. [116] used the XGBoost classifier to predict the mortality of COVID-19 patients. Furthermore, Wang et al. [89] proposed a deep convolutional neural network approach called COVID-Net to diagnose COVID-19 from radiography images. Sun et al. [117] applied the Support Vector Machine to predict severe symptoms in COVID-19 patients using four clinical indicators. The study reported an accuracy of 77.5% and a specificity of 78.4%.

Chimmula and Zhang [71] applied the Long Short Term Memory (LSTM) algorithm, a type of recurrent neural network, to forecast COVID-19 prevalence. The proposed method achieved an accuracy of 93.4% on the test set. Other studies have also focused on forecasting COVID-19 prevalence. For example, Chintalapudi et al. [118] and Gupta et al. [119] applied the Auto-Regressive Integrated Moving Average (ARIMA), a time-series method, to forecast the number of confirmed cases. Ref. [120] used a modified stacked auto-encoder method to forecast the prevalence of confirmed cases in China. A summary of articles reviewing COVID-19 diagnosis and forecasting can be found in Table 4.

### 6.3. AI for the Treatment of COVID-19

**Figure 3:**
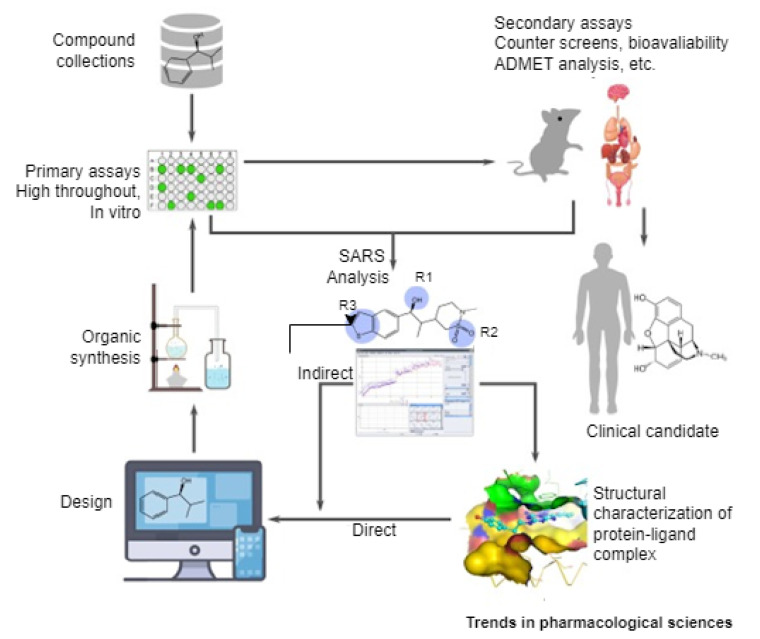
A pipeline of drug discovery and development [125].

AI is changing the landscape of many disciplines, particularly the pharmaceutical industry [121,122,123]. Through clinical trials, AI has found applications in the area of drug discovery even prior to the existence of COVID-19. Anecdotal evidence revealed that drug discovery and development are capital-intensive processes that typically cost billions of US Dollars and take about twelve years on a typical average [124,125,126]. In itself, drug discovery and development involve target selection and validation, screening of compounds identified from molecular libraries, preclinical studies, and drug candidates, which must pass clinical trials by being administered to patients (See Figure 3). Recently, this landscape has been changing with the exploration of AI to the voluminous data produced in the genomics field [121,125].

Arshadi et al. [127] outlined three approaches of AI to drug discovery: protein-based, RNA-based, and generative methods. The study showed that AI was useful in identifying unique drug and disease relationships. Furthermore, the study reviewed Benevolent AI, a UK-based organisation that integrated biomedical data from structured and unstructured sources through its AI-knowledge graph. Other organisations that have intensified efforts in using AI for COVID-19 drug discovery include Innoplexus through assessing the capability of Hydroxychloroquine and Remdesivir in the treatment of COVID-19 [128]. Similarly, Deargen and Gero used AI techniques to recommend atazanavir and niclosamide-nitazoxanide, respectively, for treating the virus [128,129,130].

In a study that utilised AI for drug screening, Delijewski and Haneczok [131] applied a supervised machine learning model based on gradient-boosting, an ensemble learning technique to identify zafirlukast as the best-repurposed candidate drug in the fight against COVID-19. The study utilised the Food and Drug Administration (FDA) set of approved drugs as the dataset, consisting of approximately 290,000 negative and 405 active molecules of COVID-19 3CL pro inhibitors, and concluded that the ML algorithm was helpful in the drug identification. Meanwhile, Pham et al. [132] proposed a graph-based neural network called DeepCE, a technique used for predicting chemical-induced gene expression profiles from chemical and biological objects. The study achieved excellent performance on the L1000 dataset and indicated that it could also be helpful for phenotype-based drug screening.

In a pathogenesis study used to find possible drug candidates, Kabra and Singh [133] used ML algorithms in a high-dimensional nucleotide sequence dataset for selecting and identifying peptides against a strain of the COVID-19 virus. The dataset consists of 2765 sequences of COVID-19 patients from different countries. The study concluded that the ML algorithms obtained excellent performance and opened a new dimension in designing and generating peptides with desired targets. Meanwhile, Jin et al. [134] proposed a drug-target interaction using a deep learning architecture, ComboNet, to predict whether a drug is likely to bind to a biological target. ComboNet consists of a graph convolutional neural network that learns a molecule representation and a linear function that learns antiviral activity and synergies in biological targets. According to the study, ComboNet performed very well despite limited drug combination training data.

### 6.4. AI for COVID-19 Surveillance

**Figure 4:**
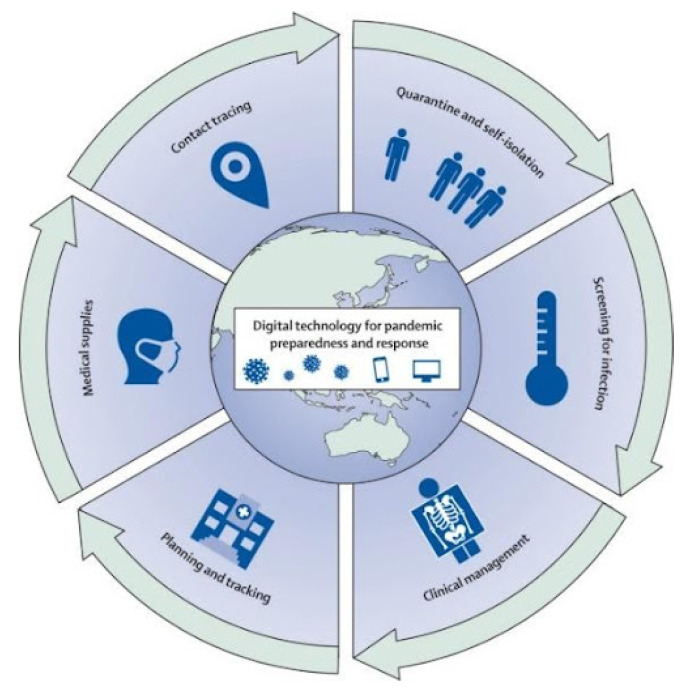
Digital tools for pandemic preparedness and response [139].

Many AI techniques have been used to build surveillance tools that predict the impact of COVID-19 [135,136,137]. As represented in Figure 4, these tools can help towards pandemic combating strategies, which can be difficult if manual methods are applied. According to Arora et al. [138], these tools incorporate several features, such as location tracking, travel data, epidemiological and behavioural patterns, to build reliable surveillance toolkits. Although these tools have been effective in reducing the spread of COVID-19, they have been widely criticised for privacy infringements.

Different machine learning models have been used to curb the spread of infection in several countries. For example, the Taiwan authorities implemented AI-based health checks for airline travellers who had visited Wuhan, China, after the outbreak was reported [140]. Taiwan attributed the low number of death cases to the AI surveillance system. Before the COVID-19 pandemic, many Chinese cities had surveillance security cameras linked to AI-based facial recognition systems [141]. When the pandemic started, these technologies were repurposed with thermal imaging for screening citizens with high temperatures.

Furthermore, South Korean CDC deployed its contact-tracing system, known as COVID-19 Smart Management System, to trace the movement of individuals with COVID-19 [142]. The system incorporated security footage, credit card records, and global positioning system (GPS) data from cell phones. Meanwhile, a US-based firm, Swedish Health Services, developed a platform for healthcare professionals to track COVID-19 cases in hospitals [139]. The app aimed to use the tracking information in allocating healthcare resources and knowing the facilities’ status. In India, a local government used geo-mapping of quarantine locations and CCTV recordings to track possible COVID-19 patients [143]. Similar technologies have been used in Israel, Argentina, and Morocco [143,144].

## 7. Discussion and Conclusions

The health and economic impact of the COVID-19 pandemic has been severe worldwide. Numerous researchers have proposed several COVID-19 diagnosis techniques. Generally, these techniques are based on antibody tests that detect the presence of proteins the body produces in response to a previous infection, molecular tests used for detecting viral genomic material, and CT tests that examine a person’s lung. Among these diagnostic techniques, the RT-PCR test that detects various regions of the SARS-CoV-1 genome is the gold standard for diagnosing COVID-19. However, the scarcity of testing tools such as RT-PCR kits could occur during pandemic emergencies. Hence, it is vital to have several testing options. Also, the RT-PCR testing technique is expensive, and many low-income countries cannot afford sufficient testing of a greater part of their population.

Meanwhile, a comprehensive review of COVID-19 diagnostic methods has been conducted in this research. The study covered current and emerging diagnostic tests, including RT-PCR, rapid antigen detection, and AI-based methods. Also, this research discussed other areas where AI has been applied to curb the spread of COVID-19, such as surveillance, tracking and dashboarding, forecasting, and treatment. Furthermore, it is necessary to state that different methods are required to control and prevent the spread of COVID-19 effectively in addition to clinical diagnosis. While highly accurate and sensitive tests are needed in the fight against COVID-19, it is also necessary to have a somewhat rapid point of care and easy to use self-testing kits.

## Figures and Tables

**Table 1:** Notable developed and emerging diagnostics deployed.

Detection System	Technology	Biomarker	Principle
Rapid antigen test	Lateral flow	Protein	Detection of Colorimetric through the use of paper with gold-coated antibodies [23]
ELISA	ELISA	Protein	The induction of virus colour change in enzymatic reaction in the presence of target antigen [24]
Biobarcode assay	DNA-mediated immunoassay	Protein	Involve the conjugation of gold nanoparticles with DNA through the help of protein signal detection [25]
Quantum dots barcode	Barcode	Nucleic acid	Capture of viral DNA and RNA through quantum beads [26]
Magnetic bead	Magnetic	Nucleic acid	Detection of PCR through the help of magnetically isolated bacteria [27]
LAMP	LAMP	Nucleic acid	Isothermal DNA synthesis through the signal of turbidity detection [28]
Smartphone dongle	ELISA	Protein	ELISA by microfluidic set up [29]
RT-LAMP	LAMP	Nucleic acid	RNA target generation through reverse transcriptase LAMP reaction [30]
CRISPR	RPA	Nucleic acid	Lateral flow nucleic assay by the help of PCR and CRISPR/Ca9 [31]
CRISPR	RT-RPA	Nucleic acid	SHERLOCK, RPA detection by multiplexed fluorescence spectroscopy [32]

**Table 2:** COVID-19 datasets available for developing ML models.

References	Dataset	Size	Image Modality	Techniques	Evaluation Result (%)
[85]	SARS-CoV-2	2482 scans (1252–positive, 1230–negative)	CT	xDNN	F1 = 97.31
[87]	LIDC		CT	Deep Learning	Acc = 90.8, Sen = 84, Spe = 93
[88]	SARS-CoV-2	2482	CT	EfficientNet	Acc = 87.6, F1 = 86.19, AUC = 90.5
[89]	COVIDx	13,975–13,870 positive patient	CXR	DCNN	Sen = 91.0
[94]	OSR, Istituto Ortopedico Galeazzi (IOG)	1925	CXR	Logistic regression, Naïve bayes, KNN, Random forest, SVM	AUC = 87, Spe = 94
[65]	COVIDx		X-ray	CNN—Capsule network	Acc = 95.7, Sen = 90, Spe = 95.8, AUC = 0.97

**Table 3:** A summary of open data dashboards for COVID-19 tracking and prediction.

References	Name	Country	Purpose	Coverage	Medium
[99]	John Hopkins CSSE	United States	Tracking and Prediction	Worldwide	Web
[98]	COVID-19 Data Hub	Canada	Tracking	Worldwide	Web
[107]	COVID-19 Tracker	United States	Tracking	Worldwide	Web
[108]	COVID-19 Dashboard	Cyprus	Tracking	Worldwide	Web
[109]	COVID-Track	United States	Tracking	Worldwide	Web
[105]	Africa CDC COVID-19	All member states	Tracking	Africa	Web
[106]	COVID-19 Open data	Panama	Tracking and Prediction	Panama	Web
[110]	α-Satellite	United States	Risk assessment	United States	Web
[111]	COVID-19 ZA South Africa	South-Africa	Tracking	South-Africa	Web
[112]	Saudi MoH COVID-19 Dashboard	Saudi Arabia	Tracking	Saudi Arabia	Web

**Table 4:** A summary of literature review of articles for COVID-19 diagnosis and forecasting.

References	Model	Scope	Evaluation Results	Datasets
[113]	Random Forest	Diagnosis	Accuracy = 96.9	Private, Blood samples
[89]	CNN	Diagnosis	Accuracy = 93.3%	Private, Chest X-ray images
[115]	XGBoost	Mortality risk prediction	Survival Accuracy = 100%, Mortality Risk = 81%	Private, Blood samples
[116]	XGBoost	Mortality risk prediction	AUC = 90% (Out of sample) AUC = 0.92 (Seville)	Private
[117]	Support Vector Machine	Prediction	Accuracy = 77.5% AUC = 78.4%	Private, Chest X-ray images
[71]	LSTM-RNN	Forecasting	Accuracy = 93.4%	Public dataset: John Hopkins and Canadian Health Authority
[119]	ARIMA	Forecasting	Accuracy = 90%	Public dataset: John Hopkins
[120]	Stacked Auto-Encoder	Forecasting	Unknown	WHO
[114]	Random Forest	Diagnosis	Accuracy = 87.5, AUC = 91%	Private, Chest X-ray images
[118]	ARIMA	Forecasting	Accuracy = 93.75%	Public dataset: Italian Ministry of Health

## Data Availability

The data presented in this study are available in article.

## References

[1] Worldometer COVID-19 Coronavirus Pandemic Weekly Coronavirus Cases. https://www.worldometers.info/coronavirus/.

[2] Sheridan C. (2020). Coronavirus and the race to distribute reliable diagnostics. Nat. Biotechnol..

[3] Corman V., Bleicker T., Brünink S., Drosten C., Landt O., Koopmans M., Zambon M., Peiris M. (2020). Diagnostic Detection of Wuhan Coronavirus 2019 by Real-Time RT-PCR.

[4] Xie X., Zhong Z., Zhao W., Zheng C., Wang F., Liu J. (2020). Chest CT for typical coronavirus disease 2019 (COVID-19) pneumonia: Relationship to negative RT-PCR testing. Radiology.

[5] WHO (2020). Laboratory Testing for Coronavirus Disease (COVID-19) in Suspected Human Cases: Interim Guidance, 19 March 2020.

[6] Guan W.J., Ni Z.Y., Hu Y., Liang W.H., Ou C.Q., He J.X., Liu L., Shan H., Lei C.L., Hui D.S. (2020). Clinical characteristics of coronavirus disease 2019 in China. N. Engl. J. Med..

[7] Lu R., Zhao X., Li J., Niu P., Yang B., Wu H., Wang W., Song H., Huang B., Zhu N. (2020). Genomic characterisation and epidemiology of 2019 novel coronavirus: Implications for virus origins and receptor binding. Lancet.

[8] Wu A., Peng Y., Huang B., Ding X., Wang X., Niu P., Meng J., Zhu Z., Zhang Z., Wang J. (2020). Genome composition and divergence of the novel coronavirus (2019-nCoV) originating in China. Cell Host Microbe.

[9] Yang Y., Yang M., Yuan J., Wang F., Wang Z., Li J., Zhang M., Xing L., Wei J., Peng L. (2020). Laboratory diagnosis and monitoring the viral shedding of SARS-CoV-2 infection. Innovation.

[10] Wu Z., McGoogan J.M. (2020). Characteristics of and important lessons from the coronavirus disease 2019 (COVID-19) outbreak in China: Summary of a report of 72 314 cases from the Chinese Center for Disease Control and Prevention. JAMA.

[11] Aruleba K., Obaido G., Ogbuokiri B., Fadaka A.O., Klein A., Adekiya T.A., Aruleba R.T. (2020). Applications of computational methods in biomedical breast cancer imaging diagnostics: A review. J. Imaging.

[12] Mienye I.D., Sun Y. (2021). Improved heart disease prediction using particle swarm optimization based stacked sparse autoencoder. Electronics.

[13] Mienye I.D., Sun Y. (2021). Performance analysis of cost-sensitive learning methods with application to imbalanced medical data. Inform. Med. Unlock..

[14] Vaishya R., Javaid M., Khan I.H., Haleem A. (2020). Artificial Intelligence (AI) applications for COVID-19 pandemic. Diabetes Metab. Syndr. Clin. Res. Rev..

[15] Kumari P., Singh A., Ngasainao M.R., Shakeel I., Kumar S., Lal S., Singhal A., Sohal S.S., Singh I.K., Hassan M.I. (2020). Potential diagnostics and therapeutic approaches in COVID-19. Clin. Chim. Acta.

[16] Kriza C., Amenta V., Zenié A., Panidis D., Chassaigne H., Urbán P., Holzwarth U., Sauer A.V., Reina V., Griesinger C.B. (2021). Artificial intelligence for imaging-based COVID-19 detection: Systematic review comparing added value of AI versus human readers. Eur. J. Radiol..

[17] Cau R., Faa G., Nardi V., Balestrieri A., Puig J., Suri J.S., SanFilippo R., Saba L. (2022). Long-COVID diagnosis: From diagnostic to advanced AI-driven models. Eur. J. Radiol..

[18] Esenogho E., Mienye I.D., Swart T.G., Aruleba K., Obaido G. (2022). A neural network ensemble with feature engineering for Improved Credit Card Fraud Detection. IEEE Access.

[19] Hassan H., Ren Z., Zhao H., Huang S., Li D., Xiang S., Kang Y., Chen S., Huang B. (2021). Review and classification of AI-enabled COVID-19 CT imaging models based on computer vision tasks. Comput. Biol. Med..

[20] Verma A., Amin S.B., Naeem M., Saha M. (2021). Detecting COVID-19 from chest computed tomography Scans using AI-Driven Android Application. arXiv.

[21] Bernheim A., Mei X., Huang M., Yang Y., Fayad Z.A., Zhang N., Diao K., Lin B., Zhu X., Li K. (2020). Chest CT findings in coronavirus disease-19 (COVID-19): Relationship to duration of infection. Radiology.

[22] World Health Organization (WHO). https://www.who.int/news/item/26-11-2021-classification-of-omicron-(b.1.1.529)-sars-cov-2-variant-of-concern.

[23] Bosch I., de Puig H., Hiley M., Carré-Camps M., Perdomo-Celis F., Narváez C.F., Salgado D.M., Senthoor D., O’Grady M., Phillips E. (2017). Rapid antigen tests for dengue virus serotypes and Zika virus in patient serum. Sci. Transl. Med..

[24] Rowe T., Abernathy R.A., Hu-Primmer J., Thompson W.W., Lu X., Lim W., Fukuda K., Cox N.J., Katz J.M. (1999). Detection of antibody to avian influenza A (H5N1) virus in human serum by using a combination of serologic assays. J. Clin. Microbiol..

[25] Thaxton C.S., Elghanian R., Thomas A.D., Stoeva S.I., Lee J.S., Smith N.D., Schaeffer A.J., Klocker H., Horninger W., Bartsch G. (2009). Nanoparticle-based bio-barcode assay redefines “undetectable” PSA and biochemical recurrence after radical prostatectomy. Proc. Natl. Acad. Sci. USA.

[26] Kim J., Biondi M.J., Feld J.J., Chan W.C. (2016). Clinical validation of quantum dot barcode diagnostic technology. ACS Nano.

[27] Nilsson H.O., Aleljung P., Nilsson I., Tyszkiewicz T., Wadström T. (1996). Immunomagnetic bead enrichment and PCR for detection of Helicobacter pylori in human stools. J. Microbiol. Methods.

[28] Imai M., Ninomiya A., Minekawa H., Notomi T., Ishizaki T., Van Tu P., Tien N.T.K., Tashiro M., Odagiri T. (2007). Rapid diagnosis of H5N1 avian influenza virus infection by newly developed influenza H5 hemagglutinin gene-specific loop-mediated isothermal amplification method. J. Virol. Methods.

[29] Laksanasopin T., Guo T.W., Nayak S., Sridhara A.A., Xie S., Olowookere O.O., Cadinu P., Meng F., Chee N.H., Kim J. (2015). A smartphone dongle for diagnosis of infectious diseases at the point of care. Sci. Transl. Med..

[30] Shirato K., Nishimura H., Saijo M., Okamoto M., Noda M., Tashiro M., Taguchi F. (2007). Diagnosis of human respiratory syncytial virus infection using reverse transcription loop-mediated isothermal amplification. J. Virol. Methods.

[31] Wang X., Xiong E., Tian T., Cheng M., Lin W., Wang H., Zhang G., Sun J., Zhou X. (2020). Clustered regularly interspaced short palindromic repeats/Cas9-mediated lateral flow nucleic acid assay. ACS Nano.

[32] Kellner M.J., Koob J.G., Gootenberg J.S., Abudayyeh O.O., Zhang F. (2019). SHERLOCK: Nucleic acid detection with CRISPR nucleases. Nat. Protoc..

[33] Tahamtan A., Ardebili A. (2020). Real-time RT-PCR in COVID-19 detection: Issues affecting the results. Expert Rev. Mol. Diagn..

[34] Udugama B., Kadhiresan P., Kozlowski H.N., Malekjahani A., Osborne M., Li V.Y., Chen H., Mubareka S., Gubbay J.B., Chan W.C. (2020). Diagnosing COVID-19: The disease and tools for detection. ACS Nano.

[35] Brihn A., Chang J., OYong K., Balter S., Terashita D., Rubin Z., Yeganeh N. (2021). Diagnostic performance of an antigen test with RT-PCR for the detection of SARS-CoV-2 in a hospital setting—Los Angeles county, California, June–August 2020. Morb. Mortal. Wkly. Rep..

[36] Reich N., Lowe C.F., Puddicombe D., Matic N., Greiner J., Simons J., Leung V., Chu T., Naik H., Myles N. (2021). Diagnostic accuracy of RT-PCR for detection of SARS-CoV-2 compared to a “composite reference standard” in hospitalized patients. medRxiv.

[37] Ferté T., Ramel V., Cazanave C., Lafon M.E., Bébéar C., Malvy D., Georges-Walryck A., Dehail P. (2021). Accuracy of COVID-19 rapid antigenic tests compared to RT-PCR in a student population: The StudyCov study. J. Clin. Virol..

[38] Khatami F., Saatchi M., Zadeh S.S.T., Aghamir Z.S., Shabestari A.N., Reis L.O., Aghamir S.M.K. (2020). A meta-analysis of accuracy and sensitivity of chest CT and RT-PCR in COVID-19 diagnosis. Sci. Rep..

[39] Jakobsen K.K., Jensen J.S., Todsen T., Kirkby N., Lippert F., Vangsted A.M., Klokker M., von Buchwald C. (2022). Accuracy of anterior nasal swab rapid antigen tests compared with RT-PCR for massive SARS-CoV-2 screening in low prevalence population. Apmis.

[40] Sethuraman N., Jeremiah S.S., Ryo A. (2020). Interpreting diagnostic tests for SARS-CoV-2. JAMA.

[41] Fazio E., Abousiam M., Caselli A., Accorona R., Nebiaj A., Ermoli I., Erckert B., Calabrese L., Gazzini L. (2020). Proper procedures for performing nasopharyngeal and oropharyngeal swabs for COVID-19. ATS Sch..

[42] Yüce M., Filiztekin E., Özkaya K.G. (2021). COVID-19 diagnosis—A review of current methods. Biosens. Bioelectron..

[43] Point-of-Care-Testing. https://www.cdc.gov/csels/dls/point-of-care-testing-risk-assessment-basics.html.

[44] Li C., Zhao C., Bao J., Tang B., Wang Y., Gu B. (2020). Laboratory diagnosis of coronavirus disease-2019 (COVID-19). Clin. Chim. Acta Int. J. Clin. Chem..

[45] Gremmels H., Winkel B.M., Schuurman R., Rosingh A., Rigter N.A., Rodriguez O., Ubijaan J., Wensing A.M., Bonten M.J., Hofstra L.M. (2021). Real-life validation of the Panbio™ COVID-19 antigen rapid test (Abbott) in community-dwelling subjects with symptoms of potential SARS-CoV-2 infection. EClinicalMedicine.

[46] Torres I., Poujois S., Albert E., Colomina J., Navarro D. (2021). Evaluation of a rapid antigen test (Panbio™ COVID-19 Ag rapid test device) for SARS-CoV-2 detection in asymptomatic close contacts of COVID-19 patients. Clin. Microbiol. Infect..

[47] Mak G.C., Lau S.S., Wong K.K., Chow N.L., Lau C., Lam E.T., Chan R.C., Tsang D.N. (2021). Evaluation of rapid antigen detection kit from the WHO Emergency Use List for detecting SARS-CoV-2. J. Clin. Virol..

[48] Merino P., Guinea J., Muñoz-Gallego I., González-Donapetry P., Galán J.C., Antona N., Cilla G., Hernáez-Crespo S., Díaz-de Tuesta J.L., Gual-de Torrella A. (2021). Multicenter evaluation of the Panbio™ COVID-19 rapid antigen-detection test for the diagnosis of SARS-CoV-2 infection. Clin. Microbiol. Infect..

[49] Ade-Ibijola A., Aruleba K. (2018). Automatic attendance capturing using histogram of oriented gradients on facial images. Proceedings of the 2018 IST-Africa Week Conference (IST-Africa).

[50] Zhuang B., Shen C., Tan M., Liu L., Reid I. Structured binary neural networks for accurate image classification and semantic segmentation. Proceedings of the IEEE/CVF Conference on Computer Vision and Pattern Recognition.

[51] Mienye I.D., Sun Y., Wang Z. (2020). Improved sparse autoencoder based artificial neural network approach for prediction of heart disease. Inform. Med. Unlock..

[52] Weiss P., Murdoch D.R. (2020). Clinical course and mortality risk of severe COVID-19. Lancet.

[53] Phua J., Weng L., Ling L., Egi M., Lim C.M., Divatia J.V., Shrestha B.R., Arabi Y.M., Ng J., Gomersall C.D. (2020). Intensive care management of coronavirus disease 2019 (COVID-19): Challenges and recommendations. Lancet Respir. Med..

[54] Mushtaq J., Pennella R., Lavalle S., Colarieti A., Steidler S., Martinenghi C.M., Palumbo D., Esposito A., Rovere-Querini P., Tresoldi M. (2021). Initial chest radiographs and artificial intelligence (AI) predict clinical outcomes in COVID-19 patients: Analysis of 697 Italian patients. Eur. Radiol..

[55] Bachtiger P., Peters N.S., Walsh S.L. (2020). Machine learning for COVID-19—Asking the right questions. Lancet Digit. Health.

[56] Rinderknecht M.D., Klopfenstein Y. (2021). Predicting critical state after COVID-19 diagnosis: Model development using a large US electronic health record dataset. NPJ Digit. Med..

[57] Roberts M., Driggs D., Thorpe M., Gilbey J., Yeung M., Ursprung S., Aviles-Rivero A.I., Etmann C., McCague C., Beer L. (2020). Machine learning for COVID-19 detection and prognostication using chest radiographs and CT scans: A systematic methodological review. arXiv.

[58] Magar R., Yadav P., Farimani A.B. (2021). Potential neutralizing antibodies discovered for novel corona virus using machine learning. Sci. Rep..

[59] Hazarika B.B., Gupta D. (2020). Modelling and forecasting of COVID-19 spread using wavelet-coupled random vector functional link networks. Appl. Soft Comput..

[60] Elaziz M.A., Hosny K.M., Salah A., Darwish M.M., Lu S., Sahlol A.T. (2020). New machine learning method for image-based diagnosis of COVID-19. PLoS ONE.

[61] Ghosh A., Nundy S., Mallick T.K. (2020). How India is dealing with COVID-19 pandemic. Sens. Int..

[62] Sujath R., Chatterjee J.M., Hassanien A.E. (2020). A machine learning forecasting model for COVID-19 pandemic in India. Stoch. Environ. Res. Risk Assess..

[63] Kushwaha S., Bahl S., Bagha A.K., Parmar K.S., Javaid M., Haleem A., Singh R.P. (2020). Significant applications of machine learning for COVID-19 pandemic. J. Ind. Integr. Manag..

[64] Pinter G., Felde I., Mosavi A., Ghamisi P., Gloaguen R. (2020). COVID-19 pandemic prediction for Hungary; a hybrid machine learning approach. Mathematics.

[65] Li L., Qin L., Xu Z., Yin Y., Wang X., Kong B., Bai J., Lu Y., Fang Z., Song Q. (2020). Artificial intelligence distinguishes COVID-19 from community acquired pneumonia on chest CT. Radiology.

[66] Khan R.S., Rehman I.U. (2020). Spectroscopy as a tool for detection and monitoring of Coronavirus (COVID-19). Expert Rev. Mol. Diagn..

[67] LeCun Y., Bengio Y., Hinton G. (2015). Deep learning. Nature.

[68] Rusk N. (2016). Deep learning. Nature Methods.

[69] Yu K., Jia L., Chen Y., Xu W. (2013). Deep learning: Yesterday, today, and tomorrow. J. Comput. Res. Dev..

[70] Zeroual A., Harrou F., Dairi A., Sun Y. (2020). Deep learning methods for forecasting COVID-19 time-Series data: A Comparative study. Chaos Solitons Fractals.

[71] Chimmula V.K.R., Zhang L. (2020). Time series forecasting of COVID-19 transmission in Canada using LSTM networks. Chaos Solitons Fractals.

[72] Asraf A., Islam M.Z., Haque M.R., Islam M.M. (2020). Deep learning applications to combat novel coronavirus (COVID-19) pandemic. SN Comput. Sci..

[73] Shan K., Ouyang T., Wang X., Yang H., Zhou B., Wu Z., Shang M. (2021). Temporal prediction of algal parameters in Three Gorges Reservoir based on highly time-resolved monitoring and long short-term memory network. J. Hydrol..

[74] Hochreiter S., Schmidhuber J. (1997). Long short-term memory. Neural Comput..

[75] Alzubaidi L., Zhang J., Humaidi A.J., Al-Dujaili A., Duan Y., Al-Shamma O., Santamaría J., Fadhel M.A., Al-Amidie M., Farhan L. (2021). Review of deep learning: Concepts, CNN architectures, challenges, applications, future directions. J. Big Data.

[76] Nasir J.A., Khan O.S., Varlamis I. (2021). Fake news detection: A hybrid CNN-RNN based deep learning approach. Int. J. Inf. Manag. Data Insights.

[77] Desai M., Shah M. (2021). An anatomization on breast cancer detection and diagnosis employing multi-layer perceptron neural network (MLP) and Convolutional neural network (CNN). Clin. eHealth.

[78] Ismael A.M., Şengür A. (2021). Deep learning approaches for COVID-19 detection based on chest X-ray images. Expert Syst. Appl..

[79] Kedia P., Anjum Katarya R. (2021). CoVNet-19: A Deep Learning model for the detection and analysis of COVID-19 patients. Appl. Soft Comput..

[80] Madhavan M.V., Khamparia A., Gupta D., Pande S., Tiwari P., Hossain M.S. (2021). Res-CovNet: An internet of medical health things driven COVID-19 framework using transfer learning. Neural Comput. Appl..

[81] Kavuran G., İn E., Geçkil A.A., Şahin M., Berber N.K. (2022). MTU-COVNet: A hybrid methodology for diagnosing the COVID-19 pneumonia with optimized features from multinet. Clin. Imaging.

[82] Mgboh U., Ogbuokiri B., Obaido G., Aruleba K. (2020). Visual Data Mining: A Comparative Analysis of Selected Datasets. International Conference on Intelligent Systems Design and Applications.

[83] Mohamadou Y., Halidou A., Kapen P.T. (2020). A review of mathematical modeling, artificial intelligence and datasets used in the study, prediction and management of COVID-19. Appl. Intell..

[84] He X., Yang X., Zhang S., Zhao J., Zhang Y., Xing E., Xie P. (2020). Sample-efficient deep learning for COVID-19 diagnosis based on CT scans. medRxiv.

[85] Angelov P., Almeida Soares E. (2020). SARS-CoV-2 CT-scan dataset: A large dataset of real patients CT scans for SARS-CoV-2 identification. medRxiv.

[86] Jaiswal A., Gianchandani N., Singh D., Kumar V., Kaur M. (2020). Classification of the COVID-19 infected patients using DenseNet201 based deep transfer learning. J. Biomol. Struct. Dyn..

[87] Harmon S.A., Sanford T.H., Xu S., Turkbey E.B., Roth H., Xu Z., Yang D., Myronenko A., Anderson V., Amalou A. (2020). Artificial intelligence for the detection of COVID-19 pneumonia on chest CT using multinational datasets. Nat. Commun..

[88] Silva P., Luz E., Silva G., Moreira G., Silva R., Lucio D., Menotti D. (2020). COVID-19 detection in CT images with deep learning: A voting-based scheme and cross-datasets analysis. Informatics Med. Unlocked.

[89] Wang L., Lin Z.Q., Wong A. (2020). Covid-net: A tailored deep convolutional neural network design for detection of covid-19 cases from chest X-ray images. Sci. Rep..

[90] Huang L., Han R., Ai T., Yu P., Kang H., Tao Q., Xia L. (2020). Serial quantitative chest CT assessment of COVID-19: A deep learning approach. Radiol. Cardiothorac. Imaging.

[91] Wang S., Kang B., Ma J., Zeng X., Xiao M., Guo J., Cai M., Yang J., Li Y., Meng X. (2021). A deep learning algorithm using CT images to screen for Corona Virus Disease (COVID-19). Eur. Radiol..

[92] Yang X., He X., Zhao J., Zhang Y., Zhang S., Xie P. (2020). COVID-CT-dataset: A CT scan dataset about COVID-19. arXiv.

[93] Afshar P., Heidarian S., Enshaei N., Naderkhani F., Rafiee M.J., Oikonomou A., Fard F.B., Samimi K., Plataniotis K.N., Mohammadi A. (2021). COVID-CT-MD, COVID-19 computed tomography scan dataset applicable in machine learning and deep learning. Sci. Data.

[94] Hall L.O., Paul R., Goldgof D.B., Goldgof G.M. (2020). Finding covid-19 from chest X-rays using deep learning on a small dataset. arXiv.

[95] Choudrie J., Banerjee S., Kotecha K., Walambe R., Karende H., Ameta J. (2021). Machine learning techniques and older adults processing of online information and misinformation: A COVID-19 study. Comput. Hum. Behav..

[96] Budd J., Miller B.S., Manning E.M., Lampos V., Zhuang M., Edelstein M., Rees G., Emery V.C., Stevens M.M., Keegan N. (2020). Digital technologies in the public-health response to COVID-19. Nat. Med..

[97] Vargo D., Zhu L., Benwell B., Yan Z. (2021). Digital technology use during COVID-19 pandemic: A rapid review. Hum. Behav. Emerg. Technol..

[98] Guidotti E., Ardia D. (2020). COVID-19 data hub. J. Open Source Softw..

[99] Dong E., Du H., Gardner L. (2020). An interactive web-based dashboard to track COVID-19 in real time. Lancet Infect. Dis..

[100] Naudé W. (2020). Artificial Intelligence against COVID-19: An Early Review.

[101] Il Yooa K., Kronenfelda B.J. (2020). An evaluation of COVID-19 dashboards from cartographic and epidemiological perspectives. Cartogr. Geogr. Inf. Sci. (CaGIS).

[102] Stiegler N., Bouchard J.P. (2020). South Africa: Challenges and successes of the COVID-19 lockdown. Annales Médico-Psychologiques, Revue Psychiatrique.

[103] Pietz J., McCoy S., Wilck J.H. (2020). Chasing John Snow: Data analytics in the COVID-19 era. Eur. J. Inf. Syst..

[104] Hassounah M., Raheel H., Alhefzi M. (2020). Digital response during the COVID-19 pandemic in Saudi Arabia. J. Med. Internet Res..

[105] For Disease Control (CDC) A.C. (2021). Africa Centre for Disease Control (CDC) Dashboard. https://africacdc.org/covid-19/.

[106] Muñoz L., Villarreal V., Nielsen M., Caballero Y., Sittón-Candanedo I., Corchado J.M. (2021). Artificial intelligence models and techniques applied to COVID-19: A review. Electronics.

[107] Patel N.V. The Best, and the Worst, of the Coronavirus Dashboards. https://www.technologyreview.com/2020/03/06/905436/best-worst-coronavirus-dashboards/.

[108] Florez H., Singh S. (2020). Online dashboard and data analysis approach for assessing COVID-19 case and death data. F1000Research.

[109] Zohner Y.E., Morris J.S. (2021). COVID-TRACK: World and USA SARS-COV-2 testing and COVID-19 tracking. BioData Min..

[110] Ye Y., Hou S., Fan Y., Qian Y., Zhang Y., Sun S., Peng Q., Laparo K. (2020). *α*-Satellite: An AI-driven System and Benchmark Datasets for Hierarchical Community-level Risk Assessment to Help Combat COVID-19. arXiv.

[111] Dsfsi.github.io (2021). COVID-19 ZA South Africa Dashboard. https://sacoronavirus.co.za/.

[112] Arabia S. (2021). COVID 19 Dashboard: Saudi Arabia. https://covid19.moh.gov.sa/.

[113] Wu J., Zhang P., Zhang L., Meng W., Li J., Tong C., Li Y., Cai J., Yang Z., Zhu J. (2020). Rapid and accurate identification of COVID-19 infection through machine learning based on clinical available blood test results. MedRxiv.

[114] Tang Z., Zhao W., Xie X., Zhong Z., Shi F., Liu J., Shen D. (2020). Severity assessment of coronavirus disease 2019 (COVID-19) using quantitative features from chest CT images. arXiv.

[115] Yan R., Zhang Y., Li Y., Xia L., Guo Y., Zhou Q. (2020). Structural basis for the recognition of SARS-CoV-2 by full-length human ACE2. Science.

[116] Bertsimas D., Lukin G., Mingardi L., Nohadani O., Orfanoudaki A., Stellato B., Wiberg H., Gonzalez-Garcia S., Parra-Calderon C.L., Robinson K. (2020). COVID-19 mortality risk assessment: An international multi-center study. PLoS ONE.

[117] Sun P., Lu X., Xu C., Sun W., Pan B. (2020). Understanding of COVID-19 based on current evidence. Journal of medical virology.

[118] Chintalapudi N., Battineni G., Amenta F. (2020). COVID-19 virus outbreak forecasting of registered and recovered cases after sixty day lockdown in Italy: A data driven model approach. J. Microbiol. Immunol. Infect..

[119] Gupta R., Pal S.K. (2020). Trend Analysis and Forecasting of COVID-19 outbreak in India. MedRxiv.

[120] Hu Z., Ge Q., Li S., Jin L., Xiong M. (2020). Artificial intelligence forecasting of covid-19 in china. arXiv.

[121] Fleming N. (2018). How artificial intelligence is changing drug discovery. Nature.

[122] Yang X., Wang Y., Byrne R., Schneider G., Yang S. (2019). Concepts of artificial intelligence for computer-assisted drug discovery. Chem. Rev..

[123] Jing Y., Bian Y., Hu Z., Wang L., Xie X.Q.S. (2018). Deep learning for drug design: An artificial intelligence paradigm for drug discovery in the big data era. AAPS J..

[124] Álvarez-Machancoses Ó., Fernández-Martínez J.L. (2019). Using artificial intelligence methods to speed up drug discovery. Expert Opin. Drug Discov..

[125] Chan H.S., Shan H., Dahoun T., Vogel H., Yuan S. (2019). Advancing drug discovery via artificial intelligence. Trends Pharmacol. Sci..

[126] Zhavoronkov A. (2018). Artificial intelligence for drug discovery, biomarker development, and Generation of Novel Chemistry. Mol. Pharm..

[127] Keshavarzi Arshadi A., Webb J., Salem M., Cruz E., Calad-Thomson S., Ghadirian N., Collins J., Diez-Cecilia E., Kelly B., Goodarzi H. (2020). Artificial intelligence for COVID-19 drug discovery and vaccine development. Front. Artif. Intell..

[128] Mohanty S., Rashid M.H.A., Mridul M., Mohanty C., Swayamsiddha S. (2020). Application of Artificial Intelligence in COVID-19 drug repurposing. Diabetes Metab. Syndr. Clin. Res. Rev..

[129] Kim H., Han G., Song J.H. (2020). A Review for Artificial Intelligence Proving to Fight Against COVID-19 Pandemic And Prefatory Health Policy. J. Med. Biomed. Appl. Sci..

[130] Mohanty C., Vinod C., Acharya S., Mahapatra N. (2022). COVID-19 drug repositioning: Present status and prospects. Modeling, Control and Drug Development for COVID-19 Outbreak Prevention.

[131] Delijewski M., Haneczok J. (2021). AI drug discovery screening for COVID-19 reveals zafirlukast as a repurposing candidate. Med. Drug Discov..

[132] Pham T.H., Qiu Y., Zeng J., Xie L., Zhang P. (2021). A deep learning framework for high-throughput mechanism-driven phenotype compound screening and its application to COVID-19 drug repurposing. Nat. Mach. Intell..

[133] Kabra R., Singh S. (2021). Evolutionary artificial intelligence based peptide discoveries for effective Covid-19 therapeutics. Biochim. Biophys. Acta (BBA) Mol. Basis Dis..

[134] Jin W., Stokes J.M., Eastman R.T., Itkin Z., Zakharov A.V., Collins J.J., Jaakkola T.S., Barzilay R. (2021). Deep learning identifies synergistic drug combinations for treating COVID-19. Proc. Natl. Acad. Sci. USA.

[135] Mbunge E. (2020). Integrating emerging technologies into COVID-19 contact tracing: Opportunities, challenges and pitfalls. Diabetes Metab. Syndr. Clin. Res. Rev..

[136] Munzert S., Selb P., Gohdes A., Stoetzer L.F., Lowe W. (2021). Tracking and promoting the usage of a COVID-19 contact tracing app. Nat. Hum. Behav..

[137] Mbunge E., Fashoto S.G., Akinnuwesi B., Metfula A., Simelane S., Ndumiso N. (2021). Ethics for integrating emerging technologies to contain COVID-19 in Zimbabwe. Hum. Behav. Emerg. Technol..

[138] Arora G., Joshi J., Mandal R.S., Shrivastava N., Virmani R., Sethi T. (2021). Artificial Intelligence in Surveillance, Diagnosis, Drug Discovery and Vaccine Development against COVID-19. Pathogens.

[139] Whitelaw S., Mamas M.A., Topol E., Van Spall H.G. (2020). Applications of digital technology in COVID-19 pandemic planning and response. Lancet Digit. Health.

[140] Wu J.T., Leung K., Leung G.M. (2020). Nowcasting and forecasting the potential domestic and international spread of the 2019-nCoV outbreak originating in Wuhan, China: A modelling study. Lancet.

[141] Chen B., Marvin S., While A. (2020). Containing COVID-19 in China: AI and the robotic restructuring of future cities. Dialogues Hum. Geogr..

[142] Lin L., Hou Z. (2020). Combat COVID-19 with artificial intelligence and big data. J. Travel Med..

[143] Do Carmo Barriga A., Martins A.F., Simões M.J., Faustino D. (2020). The COVID-19 pandemic: Yet another catalyst for governmental mass surveillance?. Soc. Sci. Humanit. Open.

[144] Calvo R.A., Deterding S., Ryan R.M. (2020). Health Surveillance during COVID-19 Pandemic. BMJ.

